# Influence of the effort-reward imbalance in college students on learning engagement: the mediating role of hope and the moderating role of growth mindset

**DOI:** 10.3389/fpsyg.2025.1650064

**Published:** 2025-10-13

**Authors:** Haixia Zhang, Qiangqiang Wang, Xinyi Xu, Shengmin Liu

**Affiliations:** ^1^School of Teacher Education, Tianshui Normal University, Tianshui, China; ^2^School of Teacher Education, Huzhou University, Huzhou, China; ^3^School of Literature, Soochow University, Suzhou, China

**Keywords:** effort-reward imbalance, hope, growth mindset, learning engagement, college students

## Abstract

**Objective:**

This study aimed to investigate the relationship between effort-reward imbalance and learning engagement among college students, as well as the mediating role of hope and the moderating role of a growth mindset.

**Method:**

A total of 665 college students participated in this study. The Effort-Reward Imbalance Scale, Hope Scale, Growth Mindset Scale, and Learning Engagement Scale were used.

**Results:**

(1) Effort-reward imbalance was significantly correlated with hope, learning engagement, and a growth mindset among college students; (2) effort-reward imbalance negatively predicted learning engagement among college students; (3) hope mediated the relationship between effort-reward imbalance and learning engagement; and (4) a growth mindset moderated the relationship between hope and learning engagement. Specifically, a stronger growth mindset mitigated the adverse effects of low hope on learning engagement among college students.

**Conclusion:**

Effort-reward imbalance influences learning engagement through the mediating role of hope and the moderating role of growth mindset. This implies that fostering a growth mindset among college students can mitigate the negative effects effort-reward imbalance and low hope on their learning engagement.

## 1 Introduction

Learning engagement is a kind of lasting, positive and complete emotional and cognitive mental state related to learning, scientific research and employment, which consists of three dimensions: vitality, dedication and absorption (Fang et al., 2008). Vitality refers to the abundant energy and strong adaptability exhibited in study and work, enabling individuals to persevere in the face of challenges. Dedication involves enthusiastically engaging in tasks, finding meaning in one’s work, and embracing challenges. Absorption refers to a high level of concentration at work, feeling like it is difficult to disengage from work and like time is passing quickly (Schaufeli et al., 2002). Learning engagement positively predicts students’ academic achievement (Furrer and Skinner, 2003; Wong et al., 2024) and mental health (Reis et al., 2015; Wang and Wang, 2024). For example, a study based on gamified learning revealed that the higher the level of students’ learning engagement was, the higher the grades they achieved (Jivani et al., 2024). These findings imply that enhancing students’ learning engagement is very important for enhancing their academic achievement and mental health. Therefore, the influencing factors and underlying mechanisms of learning engagement have become key and hot topics in educational psychology research. Unfortunately, few previous studies have investigated whether/how effort-reward imbalance predicts students’ learning engagement and the role of hope and a growth mindset in the relationship between effort-reward imbalance and learning engagement, although this imbalance is usually faced by students in school (Jin et al., 2025; Liu et al., 2024). Investigating the factors influencing learning engagement can help identify the reasons behind low levels of engagement among college students. Therefore, the aim of the present study was to examine the ability of effort-reward imbalance to predict students’ learning engagement and to investigate the role of hope and a growth mindset.

### 1.1 Effort-reward imbalance and learning engagement

The effort-reward imbalance (ERI) model posits that individuals expect their time and effort invested in work to be reciprocated with appropriate salary, respect, and career development. If individuals do not receive rewards that match their investments, they experience ERI, which adversely elicits individuals’ stress response and affects their subsequent work behaviors and health (Siegrist, 1996). For example, one recent study indicated that a greater sense of ERI could lead to greater work pressure and trigger problem drinking behaviors (Mensah et al., 2025). The ERI model can also predict the influence of the ERI students face on their learning behaviors and health. Specifically, in school education contexts, students need to invest considerable amounts of time, effort and even money to learn. They expect that their efforts will be rewarded with good achievement and recognition from others such as teachers and parents. If the rewards received by students do not match their efforts, they face ERI, which affects subsequent learning behaviors and health (Jin et al., 2025; Liu et al., 2024; Wang et al., 2023; Wege et al., 2017). According to the ERI model, when students’ rewards do not match their learning efforts, this imbalance can lead to academic stress and negative emotions such as anxiety, and all of these outcomes can have adverse effects on students’ learning engagement (Landa-Blanco et al., 2024; Lizarte Simón et al., 2024). This speculation, on the basis of the ERI model, has also been supported by empirical studies that have indicated that students who experience ERI are more prone to academic stress, negative emotions such as disappointment and depression and academic burnout (Bassanini et al., 2024; Chu and Song, 2016; Feuerhahn et al., 2012; Wu J. et al., 2021; Wu Z. et al., 2021). Research has demonstrated that students with negative academic emotions are more likely to experience attentional shifts, reduced learning flexibility, and decreased learning engagement (Derakshan et al., 2009). Consequently, ERI can predict students’ learning engagement. Recent studies examining the relationship between ERI and learning engagement among middle school students have corroborated this effect (Jin et al., 2025; Liu et al., 2024; Wu J. et al., 2021; Wu Z. et al., 2021). Therefore, hypothesis 1 was formulated.

*Hypothesis 1:* ERI negatively predicts college students’ learning engagement.

### 1.2 The mediating role of hope

Hope is an active motivational state based on an inner sense of success, which consists of three interrelated cognitive elements: goals, agency thoughts, and pathway thoughts. The goals are the direction of individual behavior and the foundation of hope; the pathway thoughts are the specific methods and plans for achieving the goal; and the agency thoughts refer to the driving force for execution, that is, the ability of an individual to recognize that they have the capacity to reach the desired goal on the basis of the existing path. It belongs to the motivational aspect of hope (Liu and Huang, 2013; Snyder, 2002). According to hope theory, the sense of inner success is the foundation for the generation of hope. Students with a high level of ERI generally experience a lower level of inner sense of success. Therefore, the ERI can negatively predict students’ hope. Although to the best of our knowledge there is no direct empirical evidence that ERI can predict hope, several studies have shown that it can predict individual psychological capital. In addition, hope is an important component of psychological capital (Feuerhahn et al., 2012; Guo et al., 2022; Han et al., 2021). Therefore, this evidence indirectly confirms the negative predictive relationship between the ERI and hope.

Hope theory indicates that people with a high level of hope tend to form more specific and feasible routes and are better at creating alternative routes. Moreover, when facing stressful situations, people with high levels of hope usually have sufficient perseverance to overcome setbacks (Liu and Huang, 2013; Snyder, 2002). According to hope theory, when students have a high level of hope in their studies, they positively and effectively engage in their studies. Thus, hope can positively predict students’ learning engagement. In addition, research has indicated that individuals with high levels of hope often possess positive goal orientations, with a focus on alternative strategies to overcome difficulties and achieve their objectives when faced with obstacles (Yavas et al., 2013). Specifically, individuals with high hope levels demonstrate greater work engagement because of their goal-oriented strategies and heightened motivation to achieve their objectives (Bakker and Demerouti, 2008). According to self-determination theory, autonomous learning motivation results in increased learning engagement and contributes to the development of long-term learning outcomes (Deci and Ryan, 2008). Hope itself is an active motivational state and thus can foster autonomous learning motivation, thereby enhancing students’ learning engagement (Deci and Ryan, 2008; Ge et al., 2025; Snyder, 2002). Therefore, hope positively predicts learning engagement. The ability of hope to predict students’ learning engagement has also been verified by several empirical studies (Azila-Gbettor et al., 2022; Chen et al., 2024; Ghbari et al., 2025; Jiang and Liu, 2024; Kit et al., 2022; Li et al., 2023). For example, Azila-Gbettor et al. (2022) examined the mediated mechanism for enhancing students’ engagement within a higher education setup via the interaction of hope and mindfulness, and the results clearly revealed that hope could significantly and positively predict student academic engagement. The above theoretical and empirical analyses clearly indicate that ERO can negatively predict individual hope, which can positively predict individual learning engagement. Therefore, hypothesis 2 can be formulated.

*Hypothesis 2:* Hope mediates the relationship between ERI and learning engagement.

### 1.3 The moderating role of growth mindset

A growth mindset, rooted in Dweck’s implicit learning theory, is a cognitive framework in which individuals believe that their intelligence and abilities can be developed through effort. Individuals with a growth mindset are inclined to embrace challenges and learn from failure. Conversely, those who have a fixed mindset view intelligence and abilities as static traits, seeking to prove themselves and avoid failure (Dweck, 2006). Achievement goal theory indicates that individuals with a growth mindset take a goal-oriented approach. They firmly believe that their abilities can be enhanced through effort and thus will keep striving toward their goals (Blackwell et al., 2007; Chazan et al., 2022; Dweck and Yeager, 2019; Heyman and Dweck, 1992; Urdan and Kaplan, 2020). According to achievement goal theory, students with a growth mindset will continue to strive and constantly increase their ability to achieve their goals even when their hope is affected by ERI. However, students with a fixed mindset may cease pursuing goals when they encounter negative events such as when their hope is affected by ERI. The findings of several experimental studies have consistently revealed a significant positive correlation between a growth mindset and learning engagement among students, with a growth mindset positively predicting learning engagement (Shida, 2024; Vestad and Bru, 2024; Zhao et al., 2021; Du, 2022). Therefore, hypothesis 3 can be formulated.

*Hypothesis 3:* A growth mindset plays a moderating role between hope and learning engagement.

**Figure 1:**
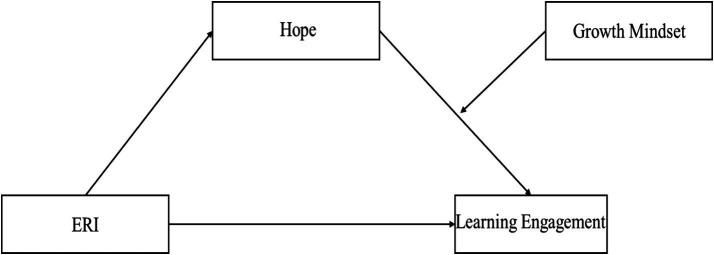
Proposed model.

In general, on the basis of the ERI model, hope theory, achievement goal theory and relevant experimental studies, the following models are derived to explain the relationship between ERI and learning engagement (Figure 1).

## 2 Methods

### 2.1 Participants and procedures

G*Power 3.1 was used to estimate the sample size needed for the present study. When the effect size was 0.15, the power level (1−*β*) was 80%, and the *α* error probability was 0.05, a minimum size of 85 participants could meet this requirement. A total of 720 college students were recruited using a convenience sampling method to participate in this questionnaire survey. Students came from different universities in Zhejiang, Jiangsu, Shanghai and other locations and were enrolled in majors including education, psychology, economics. The questionnaires were distributed to the selected students via the Questionnaire Star platform. Participants who completed the survey in significantly less time than required to thoughtfully answer all the questions (less than 90 s) were considered invalid respondents. After the invalid data were excluded, 665 valid questionnaires remained, yielding an effective response rate of 92.36%. The sample included 189 freshmen (30 males and 159 females), 128 sophomores (26 males and 102 females), 136 juniors (15 males and 121 females), and 212 seniors (33 males and 179 females), totaling 561 females and 104 males.

### 2.2 Measures

#### 2.2.1 ERI Evaluation Scale

The Effort-Reward Imbalance for Learning Scale (LERIS), developed by Fukuda et al. (2010) and revised by Chu et al. (2015), comprises two subscales: effort, with 3 items, and reward, with 4 items. The scale employs a two-point scoring system, where respondents indicate “no” or “yes” to each statement, with 1 representing “no” and 2 representing “yes.” For instance, students are asked to respond “yes” or “no” to statements such as “I will try my best to perform well in class” and “In school, I often receive encouragement from my friends.” The ERI is determined by calculating the ratio of effort to reward. The ERI ratio = effort score/(reward scores × C), where the adjustment coefficient is C (the number of items in the effort dimension/the number of items in the reward dimension). Generally, C = 0.75. This scale has been widely used in related research (Wang et al., 2023; Liu et al., 2024) and shows good reliability and validity. Confirmatory factor analysis revealed that the CFI was 0.899, RMSEA = 0.079, *χ*^2^ = 66.85, *df* = 13, *p* < 0.001.

#### 2.2.2 Hope Scale

The Hope subscale from the Positive Psychological Capital Scale developed by Zhang et al. (2010) was used. This scale consists of 6 items, with the last item of the hope dimension being a reverse-scored question. Students are asked to rate their agreement with statements such as “I am working hard to achieve my goal” and “I am pursuing my goal with confidence” on a seven-point Likert scale ranging from 1 (completely inconsistent) to 7 (completely consistent). Higher scores indicate higher levels of hope. The higher the score is, the higher the hope level of the subjects. This scale has been widely used in research (Li, 2019; Xiong et al., 2020) and indicates good reliability and validity. In this study, the Cronbach’s alpha coefficient is 0.83. Confirmatory factor analysis revealed that the CFI was 0.955, RMSEA = 0.126, *χ*^2^ = 103.996, *df* = 9, *p* < 0.001.

#### 2.2.3 Growth Mindset Scale

We utilized the Growth Mindset Scale, developed by Dweck (1990). This scale comprises six items, with items 4, 5, and 6 being reverse scored. It employs a six-point Likert scoring method, where 1 indicates “completely agree” and 6 indicates “completely disagree.” Higher scores reflect a stronger growth mindset. For example, students are asked to use an integer from 1 to 6 to judge the extent to which items such as “intelligence is hard to change” and “you can always change your intelligence to a large extent” are consistent with their actual situation. This questionnaire has been widely used in recent research (Li et al., 2025) and has demonstrated good reliability and validity. In this study, the Cronbach’s alpha coefficient is 0.80. Confirmatory factor analysis revealed that the CFI was 0.989, RMSEA = 0.062, *χ^2^* = 28.31*, df* = 8, *p* < 0.001.

#### 2.2.4 Learning Engagement Scale

We utilized the Learning Engagement Scale adapted from Schaufeli’s scale by Fang et al. (2008). This scale consists of three dimensions: focus (6 items), vitality (6 items), and dedication (5 items). It employs a seven-point Likert scoring method, where respondents rate their agreement with each statement on a scale from 1 (never) to 7 (always). Higher scores indicate a higher level of learning engagement. For example, students are asked to use an integer from 1 to 7 to judge the degree of conformity between “when I get up in the morning, I am willing to study” and “when I study, I feel energetic” and their actual situation. This questionnaire has been widely used in research (Agormedah, 2025; Liu et al., 2024) and indicates good reliability and validity. In this study, the Cronbach’s alpha coefficient was 0.95. Confirmatory factor analysis revealed that the CFI was 0.955, RMSEA = 0.066, *χ*^2^ = 449.55, *df* = 116, *p* < 0.001.

## 3 Data processing and analysis

Considering that the PROCESS plugin created by Hayes (2017) is an effective tool for establishing structural equation models used to examine the mechanisms of interaction among multiple variables and has been widely used in many studies (Liu et al., 2024; Jin et al., 2025), SPSS 26.0 and PROCESS plugin were used to analyze the collected data, and the Pearson correlation method was used to analyze the correlation between the four core variables. The bootstrap method was used to test the mediating effect. A total of 5,000 sampling times were used, and a 95% confidence interval was adopted.

### 3.1 Common method bias test

This survey collects data entirely through self-report methods to prevent common method bias caused by a single data collection method from affecting the survey results. We first conducted a common method bias test using Harman’s single-factor test with SPSS 26.0. The results revealed that 7 factors had eigenvalues greater than 1, and the first factor, before rotation, explained 36.19% of the variance, which is less than the critical standard of 40%, indicating that there is no severe common method bias in this study (Zhou and Long, 2004).

### 3.2 Descriptive statistics and correlation analysis of variables

SPSS 26.0 was used for descriptive statistics and correlation analysis of ERI, hope, growth mindset, and learning engagement. The results indicated that there were significant correlations among ERI, hope, growth mindset, and learning engagement. Specifically, ERI was significantly negatively correlated with learning engagement, hope, and a growth mindset; hope was significantly positively correlated with learning engagement and a growth mindset; and a growth mindset was significantly positively correlated with learning engagement (see Table 1 for details). The correlations between each pair of variables were significant, indicating that conducting a further analysis of the mediating effect was appropriate.

**Table 1:** Descriptive statistics and correlation analysis of each variable.

Variable	*M*	SD	1	2	3	4
1. ERI rate	0.91	0.21	1.00			
2. Hope	5.04	1.02	−0.22**	1.00		
3. Growth mindset	3.22	0.85	−0.11**	0.17**	1.00	
4. Learning engagement	4.66	1.11	−0.17**	0.70**	0.27**	1.00

** Indicates *p* < 0.01; all values are rounded to two decimal places; ERI, effort-reward imbalance.

### 3.3 Multicollinearity test

In order to prevent the influence of the synonymous situation of variables from contaminating the research results, it is necessary to ensure the rationality and feasibility of the results through multicollinearity test. The multicollinearity problem is determined by the value of the variance inflation factor (Baron and Kenny, 1986). Through SPSS26.0, the data analysis shows that the variance inflation factor value of each variable does not exceed 2 and far less than 10 (Table 2), indicating that there is no serious multicollinearity problem, and the regression model test can be carried out.

**Table 2:** Result of multicollinearity test.

Variable	Non-standardized coefficient		Standardized coefficient	*t*	*p*	VIF
	B	Standard error	Beta			
Constant	0.352	0.251		1.404	0.161	
Effort–reward imbalance	0.151	0.121	−0.01	−0.371	0.711	1.055
Hope	0.031	0.045	0.671	23.864	0.000	1.074
Growth mindset	0.036	0.037	0.156	5.666	0.000	1.037

### 3.4 The mediating role of hope

The mediating effect of hope on the relationship between ERI and learning engagement was examined using Model 4 in PROCESS (Hayes, 2017). Bootstrapping with 5,000 resamples was used to determine the significance of the mediating effect and to calculate the 95% confidence interval. After controlling for gender and grade, the results revealed that ERI could significantly negatively predict hope (*β* = −0.210, *t* = −5.575, *p* < 0.01; 95% CI = [−1.410, −0.676]) and significantly negatively predict learning engagement (*β* = −0.167, *t* = −4.380, *p* < 0.01; 95% CI = [−1.313, −0.500]). Hypothesis 1 posited that ERI can predict students’ learning engagement, and a simple mediation effect test revealed that ERI had a negative effect on students’ learning engagement. These results are consistent with the prediction of Hypothesis 1; which was thus confirmed.

After we included hope as a mediator and controlled for gender and grade, the analysis revealed that the negative predictive effect of effort-reward imbalance on hope remained significant (*β* = −0.210, *t* = −5.575, *p* < 0.01; 95% CI = [−1.410, −0.676]), whereas its negative predictive effect on learning engagement was not significant (*β* = −0.022, *t* = −0.763, *p* > 0.05; 95% CI = [−0.422, 0.186]). Moreover, hope significantly positively predicted learning engagement (*β* = 0.691, *t* = 24.033, *p* < 0.01; 95% CI = [0.694, 0.818]). The above results preliminarily indicate that hope plays a full mediating role between effort-reward imbalance and learning engagement (see Table 3).

**Table 3:** The mediating role of hope.

Result variables	Predictors	*R*	*R* ^2^	*F*	*Β*	LLCI	ULCI	*t*
Hope	ERI	0.260	0.068	16.018	−0.210	−1.410	−0.676	−5.575**
	Gender				0.035	−0.109	0.305	0.928
	Grade				0.140	0.056	0.180	3.725***
Learning engagement	ERI	0.214	0.05	10.56	−0.167	−1.313	−0.500	−4.380**
	Gender				0.038	−0.115	0.344	0.983
	Grade				0.120	0.041	0.179	3.144**
Learning engagement	ERI	0.700	0.491	159.230	−0.022	−0.422	0.186	−0.763
	Hope				0.691	0.694	0.818	24.033**
	Gender				0.013	−0.127	0.208	0.477
	Grade				0.023	−0.030	0.072	0.811

** Indicates *p* < 0.01; *** indicates *p* < 0.001.

**Figure 2:**
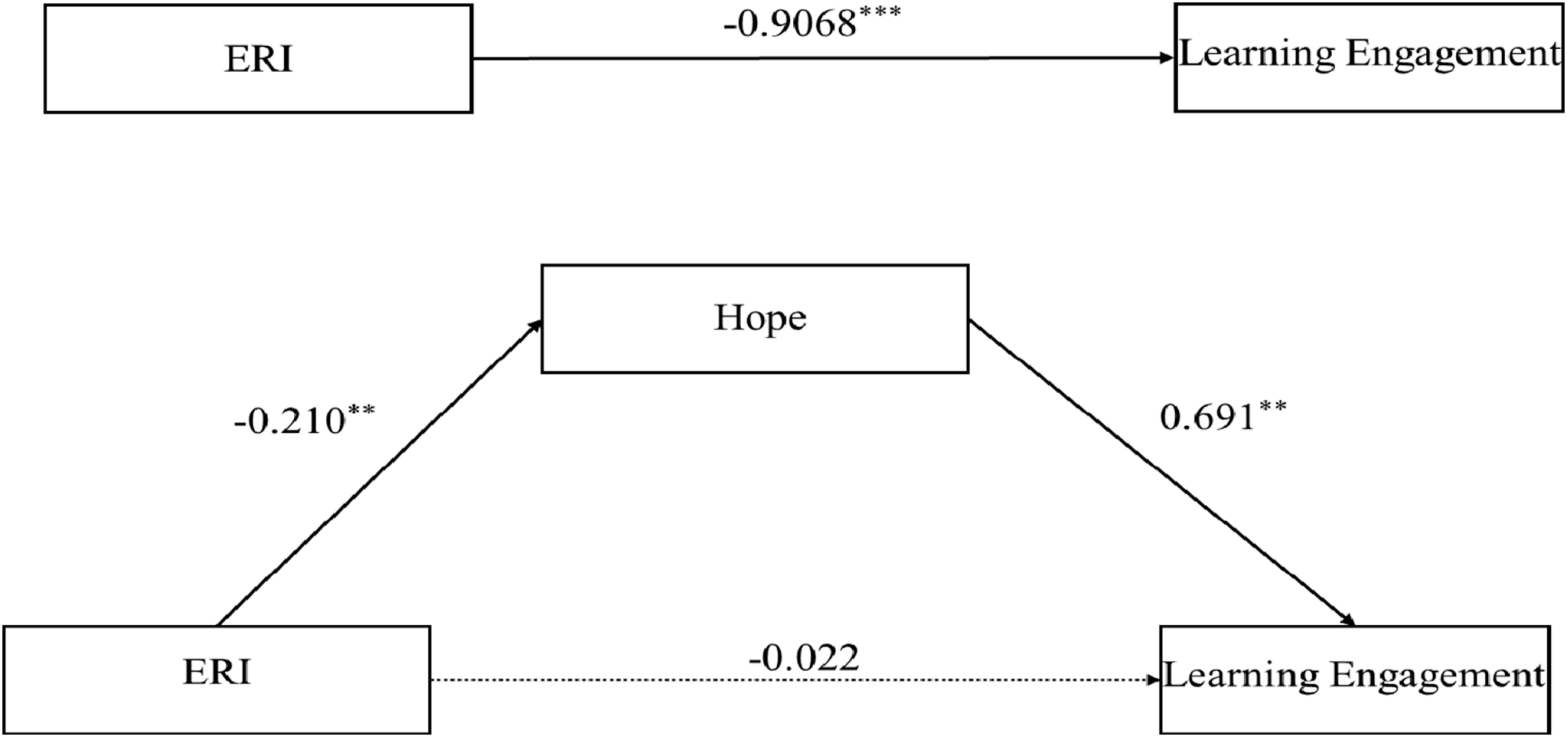
Mediating model of hope, ** indicates *p* < 0.01; *** indicates *p* < 0.001.

In the mediation model composed of ERI as the independent variable, learning engagement was the dependent variable, and hope was the mediating variable, with two paths included: the direct path from ERI to learning engagement, and the indirect path from ERI to learning engagement through hope. To further test the complete mediating effect of hope on the relationship between ERI and learning engagement, we further analyzed both the direct and indirect paths. The results showed that the direct effect of ERI on learning engagement was −0.1182 (95% CI = [−0.4222, 0.1858]). The indirect effect of ERI on learning engagement through hope was −0.7886 (95% CI = [−1.0717, −0.5127]). The confidence interval of the direct effect clearly included 0, whereas the confidence interval of the indirect effect did not include 0. This finding indicated that the path through which ERI indirectly affects learning engagement via hope is statistically significant. The results fully demonstrated that hope played a complete mediating role in the relationship between ERI and learning engagement. Hypothesis 2 speculated that hope has a mediating effect on the relationship between ERI and learning engagement. The mediation effect test fully confirmed the mediating role of hope and Hypothesis 2 was validated (see Table 4; Figure 2).

**Table 4:** The mediating role of hope.

Variable	Effect	S.E.	*p*	95% CI		Relative effect size
				LLCI	ULCI	
Total effect	−0.9068	0.2071	0.0000	−1.3134	−0.5003	
Direct effect	−0.1182	0.1548	0.4455	−0.4222	0.1858	13.03%
Indirect effect	−0.7886	0.1409	/	−1.0717	−0.5127	86.97%

### 3.5 The moderating role of growth mindset

Model 14 in PROCESS, programmed by Hayes, was used to test whether a growth mindset moderated the mediating effect of hope in the relationship between ERI and learning engagement. Bootstrapping with 5,000 resamples was employed. After controlling for gender and grade, the results revealed that hope can significantly positively predict learning engagement (*β* = 0.7343, *t* = −4.8862, *p* < 0.001; 95% CI = [0.6731, 0.7956]), the prediction of the interaction between hope and a growth mindset on learning engagement is also significant (*β* = 0.0671, *t* = 2.0374, *p* < 0.05; 95% CI = [0.0024, 0.1318]). These results indicating that a growth mindset moderated the mediating effect of hope between ERI and learning engagement (see Table 5). Further analysis showed that the effect size of the moderation is 0.0031, *F* = 4.27, *p* < 0.05, this result further stressed the moderation effect of the growth mindset on the mediating effect of hope between ERI and learning engagement.

**Table 5:** Moderation effect test (*n* = 665).

Variable	Dependent variable: hope			Dependent variable: learning engagement		
	*Β*	*t*	95% CI	*β*	*t*	95% CI
Gender	0.0979	0.9280	[−0.1093, 0.3051]	0.0440	0.5275	[−0.1197, 0.2076]
Grade	0.1181	3.7252***	[0.0559, 0.1804]	0.0264	1.0460	[−0.0232, 0.0761]
ERI	−1.0430	−5.5751***	[−1.4103, −0.6756]	−0.0686	−0.4523	[−0.3663, 0.2292]
Growth mindset	0.1937	5.2860***	[0.1217, 0.2656]			
Hope × Growth mindset	0.0671	2.0374*	[0.0024, 0.1318]			
Hope				0.7343	23.5346***	[0.6731, 0.7956]
*R* ^2^	0.0678			0.5182		
*F*	16.0178***			117.9306 ***		

* Indicates *p* < 0.05; ** indicates *p* < 0.01; *** indicates *p* < 0.001.

**Figure 3:**
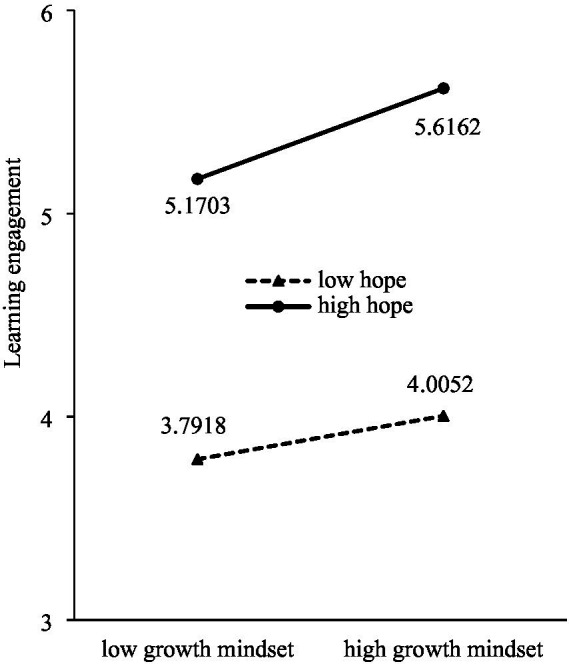
The moderating role of growth mindset.

To further examine the nature of the interaction between hope and growth mindset, we used simple slope tests to analyze the impact of hope on learning engagement for both high and low groups of individuals with a growth mindset. The results revealed that although learning engagement increased with increasing hope, the impact of hope on learning engagement differed between the low-growth mindset and high-growth mindset conditions. Specifically, in the low-growth mindset condition, the prediction of hope on learning engagement was significant (simple slope = 0.6772, *t* = 17.00, *p* < 0.001), and when the hope level increased from low to high, the increase in learning engagement was relatively small (1.3785); whereas in the high-growth mindset condition, the prediction of hope on learning engagement was also significant (simple slope = 0.7914, *t* = 18.01, *p* < 0.001), and when the hope level increased from low to high, the increase in learning engagement was relatively large (1.611). The increase in the rate of learning engagement with increasing hope in the high-growth mindset condition was clearly faster than that in the low-growth mindset condition, indicating that the ability of hope to predict learning engagement was moderated by the level of the growth mindset (Figure 3). Hypothesis 3 speculated that a growth mindset moderates the positive predictive effect of hope on learning engagement. A moderation model test revealed that having a growth mindset moderated the impact of hope on learning engagement and Hypothesis 3 was validated.

## 4 Discussion

Considering that few previous studies have investigated whether and how ERI predicts students’ learning engagement, the present study examined the prediction mechanism through which ERI affects learning engagement from the perspective of hope and individual growth mindset. On the basis of the ERI model, we deduced that when students experience ERI in their study, this ERI leads to academic stress and some negative emotions, such as anxiety, and all of these negative outcomes weaken students’ learning engagement. This study indeed revealed that ERI has a significant negative predictive effect on learning engagement. These findings support the ERI model and verify the relationship between ERI and learning engagement (Jin et al., 2025). These results enrich our understanding of the factors influencing students’ learning engagement and remind us that we should pay attention to the ERI experienced by students and its potential negative impact on their learning engagement in school education. After verifying the ability of ERI to predict learning engagement, we focused on the mediating role of hope in the relationship between ERI and learning engagement and the moderating role of growth mindset. Next, we discuss the results of this study in depth from the perspective of the mediating effect of hope and the moderation effect of a growth mindset.

### 4.1 The mediating role of hope

An analysis of the mediating effect revealed that hope played a full mediating role in the relationship between ERI and learning engagement. On the basis of hope theory and self-determination theory and many related studies, we deduced that hope can mediate the effect of ERI on students’ learning engagement. Our finding of a full mediating effect of hope in relation to ERI verified the negative impact of this imbalance on individual hope and the influence of hope on individual learning engagement. The results further supported hope theory and self-determination theory (Deci and Ryan, 2008; Liu and Huang, 2013; Snyder, 2002).

To our surprise, when hope was excluded as a mediating variable, the results showed that ERI could significantly predict individual learning engagement. These findings were consistent with the prediction of the ERI model. However, when hope was included as a mediating variable, the direct effect of ERI on individual learning engagement was not significant. Under these conditions, ERI could predict individual hope and indirectly predict individual learning engagement. These findings imply that the direct influence of ERI on learning engagement was inhibited by the mediating effect of hope. The reason may be that the influence of ERI on hope and the influence of hope on learning engagement are stronger than the influence of ERI on learning engagement. Therefore, when hope was included as a mediating variable, the direct effect of ERI on learning engagement was inhibited by the indirect mediating effect of hope on the relationship between ERI and learning engagement. A correlation analysis of the variables revealed that the correlation coefficient between ERI and hope was 0.22, the correlation coefficient between hope and learning engagement was 0.70, and the correlation coefficient between ERI and learning engagement was 0.17. Although a significant correlation itself does not prove the establishment of a causal relationship, it can, to a certain extent, provide evidence for a causal relationship. To explain the full mediating effect of hope on the relationship between ERI and learning engagement, we hypothesized that the influence of ERI on hope and the influence of hope on learning engagement was greater than the influence of ERI on hope. The correlation coefficient between these variables also supports our hypothesis.

Although several studies have investigated the mechanism underlying the effect of ERI on learning engagement (Jin et al., 2025; Liu et al., 2024), no study has investigated how ERI influences students’ learning engagement from the perspective of the mediating role of hope; thus, these results can further enrich and deepen the studies on the prediction mechanism of ERI on learning engagement. The verification of the mediating effect of hope on the relationship between ERI and learning engagement also provides important inspiration for school education. When students are in school, we can pay attention to the methods and approaches of education, enhance students’ learning efficiency, treat every student fairly, and reduce the experience of ERI for students and further reduce the negative impact of ERI on students’ hope and their learning engagement. On the other hand, we can also increase students’ hope with effort and prevent the negative impact of low hope levels on learning engagement.

### 4.2 The moderating role of growth mindset

After verifying the mediating effect of hope on the relationship between ERI and learning engagement, we investigated the moderating effect of having a growth mindset on the relationship between hope and learning engagement. The results revealed that the ability of hope to predict learning engagement was moderated by the level of a growth mindset. Specifically, the rate of increase in learning engagement with increasing hope in the high-growth mindset condition was faster than that in the low-growth mindset condition. According to achievement goal theory, we deduced that compared with students with a low-level growth mindset, students with a high-level growth mindset will continue to strive and constantly increase their ability to achieve their goals, thus moderating the impact of hope on their learning engagement. The results of the moderating role analysis clarified the moderation effect of a growth mindset, verified the deduction of achievement goal theory and provided empirical evidence for achievement goal theory. The findings concerning the moderation effect of having a growth mindset also further clarified the mechanism underlying the relationships among hope, having a growth mindset, and learning engagement and provided notable insights into school education. Specifically, when students are in school, in addition to caring about their academic achievements and ERI, we should also actively cultivate students’ growth mindset. This cognitive framework enables students to better and more calmly address the setbacks and challenges in learning.

## 5 Limitations and future research

The present study examined how ERI influences the learning engagement of students from the perspective of hope and a growth mindset. The results showed that ERI influences students’ learning engagement fully through decreasing their hope. Moreover, a growth mindset can buffer the negative impact of hope on learning engagement and thus buffer the impact of ERI on students’ learning engagement. The results of this study contribute to research on the relationship between ERI and learning engagement and fill the gap in understanding how this imbalance influences learning engagement from the perspective of hope. This study had several limitations. First, the data were collected online by a convenient sampling method, and most of the participants were females. Although the results fit our hypothesis well, the external validity of the results is limited because of the limitations of the convenient sampling method and the unbalanced gender ratio. Therefore, this study requires further testing among a larger group of college students. Second, cross-sectional data were collected to fit the proposed model on the prediction mechanism of ERI on students’ learning engagement. Because they are not as convincing as longitudinal data are, subsequent studies can further explore the causal relationships among variables using longitudinal data or design-related experiments, which would better reveal the influence of ERI on learning engagement. Finally, only the influence of ERI on learning engagement from the dimensions of hope and a growth mindset was analyzed. Subsequent studies should investigate the influence of the ERI on learning engagement from other dimensions.

## 6 Conclusion

This study examined the effects of ERI on learning engagement, the mediating role of hope and the moderating mechanism of a growth mindset. According to the study results, the following conclusions can be drawn: (1) Hope mediates the relationship between ERI and learning engagement, and (2) growth mindset plays a moderating role between hope and learning engagement. The results further revealed the prediction mechanism of ERI from the perspectives of hope and growth mindset. These results imply that educators should pay attention to reducing the occurrence of students’ ERI to prevent negative impacts on their learning engagement. In addition, educators should cultivate a growth mindset for students.

## Data Availability

The original contributions presented in the study are included in the article/supplementary material, further inquiries can be directed to the corresponding author.
